# Neural correlates of affective task switching and asymmetric affective task switching costs

**DOI:** 10.1093/scan/nsac054

**Published:** 2022-10-13

**Authors:** Cindy Eckart, Dominik Kraft, Lena Rademacher, Christian J Fiebach

**Affiliations:** Department of Psychology, Goethe University Frankfurt, Frankfurt am Main 60323, Germany; Department of Psychiatry, Psychosomatic Medicine and Psychotherapy, University Hospital, Goethe University, Frankfurt am Main 60528, Germany; Department of Psychology, Goethe University Frankfurt, Frankfurt am Main 60323, Germany; Department of Psychiatry and Psychotherapy, Tübingen Center for Mental Health, University of Tübingen, Tübingen 72076, Germany; Department of Psychology, Goethe University Frankfurt, Frankfurt am Main 60323, Germany; Department of Psychiatry and Psychotherapy, University of Lübeck, Lübeck 23562, Germany; Department of Psychology, Goethe University Frankfurt, Frankfurt am Main 60323, Germany; Brain Imaging Center, Goethe University Frankfurt, Frankfurt am Main 60528, Germany

**Keywords:** emotion, task switching, affective flexibility, asymmetric switch costs, functional MRI

## Abstract

The control of emotions is of potentially great clinical relevance. Accordingly, there has been increasing interest in understanding the cognitive mechanisms underlying the ability to switch efficiently between the processing of affective and non-affective information. Reports of asymmetrically increased switch costs when switching toward the more salient emotion task indicate specific demands in the flexible control of emotion. The neural mechanisms underlying affective task switching, however, are so far not fully understood. Using functional Magnetic Resonance Imaging (MRI) (*N* = 57), we observed that affective task switching was accompanied by increased activity in domain-general fronto-parietal control systems. Blood-oxygen-level-dependent (BOLD) activity in the posterior medial frontal and anterolateral prefrontal cortex was directly related to affective switch costs, indicating that these regions play a particular role in individual differences in (affective) task-switching ability. Asymmetric switch costs were associated with increased activity in the right inferior frontal and dorsal anterior medial prefrontal cortex, two brain regions critical for response inhibition. This suggests that asymmetric switch costs might—to a great extent—reflect higher demands on inhibitory control of the dominant emotion task. These results contribute to a refined understanding of brain systems for the flexible control of emotions and thereby identify valuable target systems for future clinical research.

## Introduction

Goal-directed behavior requires flexible switching between competing response tendencies, an ability that is in experimental psychology often operationalized using task-switching paradigms. Such paradigms most often involve switching between two different tasks conducted with the same stimuli (e.g. Schneider and Logan, 2005). Instructed task switching is consistently accompanied by declines in behavioral performance (switch costs; Monsell, 2003) and relies on a domain-general network of brain regions including the posterior parietal and dorsolateral prefrontal cortices as well as activity along the inferior frontal sulcus (Kim *et al.*, 2012; Richter and Yeung, 2014; Dajani and Uddin, 2015; Uddin, 2021). However, while competing cognitive demands from the environment are rarely as controlled as in the laboratory, they often differ in their personal relevance and affective-motivational salience. The ability to flexibly engage and disengage from emotional experiences relates to coping with stress and may thus contribute to vulnerability for stress-related affective disorders (e.g. Southwick and Charney, 2012). Accordingly, there has been an increasing interest in studying flexible behavior in the face of affective stimuli and tasks (‘affective flexibility’). Initial research indicates that ‘affective task switching’ is related to individual differences in emotion regulation (Malooly *et al.*, 2013), rumination (Genet *et al.*, 2013) and psychological resilience (Genet and Siemer, 2011) and that the efficiency of task switching varies depending on the affective nature of the task. Specifically, response time (RT) switch costs are prolonged when switching away from an affectively neutral task and toward the more salient affective task (Reeck and Egner, 2015; Kraft *et al.*, 2020; Eckart *et al.*, 2021), an imbalance resembling asymmetric switch costs between more and less dominant tasks in other contexts (e.g. Monsell *et al.*, 2000).

There is an emerging consensus that cognitive and affective brain systems do not operate in isolation but rather interactively (e.g. Pessoa, 2008; Shackman *et al.*, 2011; Okon-Singer *et al.*, 2015). Accordingly, brain regions involved in (affectively neutral) task switching may be equally responsible for task switching in the face of affective information. Indeed, previous investigations identified similar brain mechanisms for affective as for affectively neutral task switching, including lateral prefrontal and posterior parietal cortices (Piguet *et al.*, 2013; Reeck and Egner, 2015). One further region within this network, the medial frontal cortex (MFC), has been suggested as integrating negative affect and cognitive control (Shackman *et al.*, 2011) and is engaged during the interaction of emotion and cognition (Cromheeke and Mueller, 2014). However, data on the exact neuronal underpinnings of affective task switching are still scarce. Also, previous investigations (Piguet *et al.*, 2013; Reeck and Egner, 2015) did not explicitly target the neuronal correlates of asymmetric switch costs, so that their underlying neurocognitive mechanisms remain to be specified.

To study affective task switching, we have recently established a paradigm with excellent psychometric properties (Eckart *et al.*, 2021). Participants alternate between judging the emotional expression and an (affectively neutral) gender judgment, on images of faces. In the present study, we used this paradigm to replicate (a) BOLD activation results for affective task switching (Piguet *et al.*, 2013; Reeck and Egner, 2015) and (b) potential differences in the engagement of brain systems between the affective *vs* neutral tasks (independent of repetition or switch trials), in an independent study with high statistical power. Furthermore, we examined (c) the relationship between the efficiency of affective task switching (i.e. RT switch costs) and brain activation, by correlating this behavioral parameter with switching-related BOLD activation and (d) the brain mechanisms underlying asymmetric affective switch costs, by exploring the interaction between the switching condition and (affective *vs* neutral) task.

## Methods

The current study reports data and findings generated in the context of a more extensive pre-registered study investigating neuronal activation differences and commonalities among three different task switching paradigms (see https://osf.io/a64jn for the pre-registration). Sample size was chosen to allow for adequately powered correlation tests (between different task-switching paradigms) assuming effects of moderate size. Simulation results suggest that 55 subjects are necessary to achieve a power level of 0.80 for brain-behavior correlations of moderate strength (here, *r* = 0.37; cf. Yarkoni and Braver, 2010). Here, only data from one paradigm will be reported.

### Participants

In addition to common MRI exclusion criteria, we assured the absence of chronic drug abuse or medication, handedness scores below +50 in the Edinburgh Handedness Inventory (Dragovic, 2004), past head trauma or fainting, and current major medical, neurological, or psychiatric disorders (assessed with the Mini-International Neuropsychiatric Interview (M.I.N.I.); Ackenheil *et al.*, 1999). Of the total sample of 69 healthy, right-handed participants, eight dropped out during study appointments, one was excluded because of trigger problems and three participants were excluded due to excessive motion (see below), leaving a final sample of *N* = 57 (29 males, one diverse; 18 to 34 years, mean 23.96 ± 3.60). All participants gave informed consent according to a protocol approved by the local ethics committee (Proposal ID: 2018–49a, amendment to 2016–13a-c).

### Study procedure

Functional MRI (fMRI) data acquisition was performed on two appointments (interval 1–10 days, mean 3.83 ± 2.32). Assignment of experimental tasks to the first *vs* second appointment was counterbalanced. The affective switching paradigm was always preceded by a resting-state scan within its session while anatomical images were collected in the other session. Pre-experiment training included two blocks in which the tasks were trained separately. Subsequently, a mixed task-switching block was repeated until a criterion of ≥60% accuracy was reached. As current mood might have an impact on affective task switching (Grol and De Raedt, 2018), participants filled out state versions of the Positive and Negative Affect Schedule (PANAS; Krohne *et al.*, 1996) and the State-Trait Anxiety Inventory (STAI; Laux *et al.*, 2013) before scanning. The task was presented using Presentation software (Version 20.01 12.04.17 build, Neurobehavioral Systems, Inc., Berkeley, CA) on a NordicNeuroLab monitor (40”, 1920 × 1080, 60 Hz) viewed via a mirror attached above the head coil. Responses were given with the index and middle fingers of both hands and logged with a MR-compatible four-button response box (Current Designs Inc., Philadelphia, USA).

### Affective task-switching paradigm

**Fig. 1.**
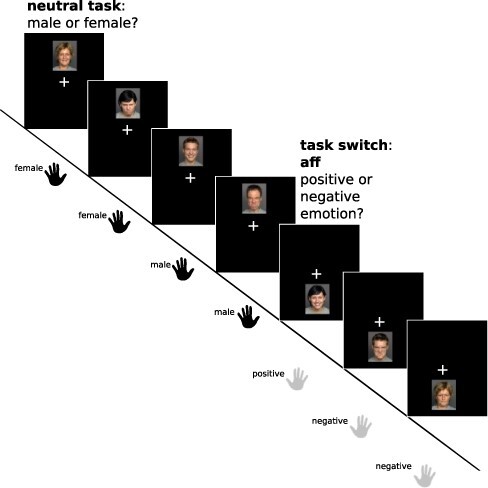
Illustration of the affective flexibility paradigm. Participants judge affective facial stimuli according to two different task rules, i.e. an affectively neutral gender task (female *vs* male) or an emotion task (happy *vs* angry facial expression). Target images were presented either above or below the central fixation cross. Location of the images changed every 3–7 trials, thereby indicating a task switch. As task rules were assigned to the two response hands, a task switch was always accompanied by a switch of response hand, and hand task assignment was counterbalanced across participants. This figure was originally published in Kraft *et al*. (2020) under a CC-BY 4.0 license.

Pictures of 60 males and 60 females with either happy or angry expression were presented, and participants performed either a gender (female *vs* male; neutral task) or emotion judgment (positive vs. negative emotional expression), depending on the location of the image (above or below a central fixation cross; see Kraft *et al.*, 2020, for further details on stimuli and procedures). Assignment of task rules to response hands and rule-to-position mapping (above *vs* below fixation) were counterbalanced across subjects. After three to seven repeat trials, the location of the target image changed, indicating a task switch and change of response hand (Figure 1). Four pseudo-randomized stimulus sequences were generated, thereby excluding direct repetitions of the same identity and assuring that the number of consecutive repeat trials between two task switches does not repeat more than twice (e.g. when two task switches were separated by five repeat trials, this number of repeat trials did not repeat more than twice). Two runs with 240 trials each were completed, of which 48 were switch trials (balanced for task, emotion and gender). Stimuli were presented for 1 s, followed by an inter-trial interval (ITI) of at least 1 s during which the fixation cross re-appeared; responses were registered for a maximum of 2 s. Following a procedure described by Liston *et al*. (2006), ITIs of varying lengths (1–13 s; mean 5.5 s) were introduced following the 48 switch trials and 48 pseudo-randomly selected repeat trials (balanced for task, emotion and gender), which were used for calculating fMRI contrasts. This was done (i) to avoid previously demonstrated effects of slower (Wodka *et al.*, 2009) and jittered (Liston *et al.*, 2006) trial sequences on behavioral switch effects and (ii) to control the total length of the experiment. Task duration was 35 minutes, including two breaks of 40s per run.

### Behavioral analyses

No participant had to be excluded due to pre-registered performance criteria [criterion: error rates (ERs >30%) in any of the two repeat conditions]. Statistical analyses were conducted in RStudio (version 1.4.1106) running on R 4.0.5 (2021–03-31; R Core Team, 2021). Trials with incorrect responses or RTs < 250 ms or greater than three standard deviations (SDs) above the person’s mean of the respective condition (after excluding error trials; 4.07 ± 4.09 trials; range: 0–23 trials) were excluded from RT analyses. RTs were slower in repeat trials directly following a temporal jitter, in accordance with previous reports of a diminished preparedness to respond in trials preceded by strong temporal jitter (Wodka *et al.*, 2009). ER did not differ significantly (see the Supplementary Materials for details). To avoid biases in our behavioral results, ER and RT analyses were restricted to the same repeat trials as selected for fMRI analyses (see above). To exclude the possibility that the selection of trials could have distorted our results, we repeated the behavioral analyses on the full set of trials, which, however, did not change the pattern of results (see the Supplementary Materials).

Selecting a subsample of trials can pose a potential problem for the interpretation of ER-based switch costs, as their reliability decreases considerably when trial numbers are reduced (e.g. Eckart *et al.*, 2021). Accordingly, behavioral analyses were focused on RT-based switch costs, which deviate from the pre-registration. Switch costs were calculated as the RT difference between the means over switch and repeat trials, separately for each task (emotion *vs* gender) and participant. A metric RT measure of asymmetric switch costs was calculated by subtracting the RT switch cost in the gender task from the RT switch cost in the emotion task. As a measure of the effect size, we calculated Hedges’ *g* for task-specific differences between repeat and switch trials. Internal consistency of switch costs (for RT but for descriptive reasons also for ERs) are reported as Spearman-Brown corrected correlations (*r*_SB_) calculated via permutation-based split-half reliability estimates (5 000 random splits) using the *splithalf* package for R (version 0.7.2; Parsons, 2020). Effects of task and condition (repeat *vs* switch) on RTs were evaluated with a 2 × 2 repeated-measures analysis of variance (ANOVA). Subsequent post hoc contrasts were calculated using R’s *emmeans* package (version 1.6.0; Lenth *et al.*, 2021), and estimated marginal means (EMMs), corresponding standard errors (SEs) and 95% confidence intervals (CIs) are reported. Potential influences of mood state on task switching were explored using Spearman correlations and by including mood scales as covariates into ANOVAs. As mood scores were intercorrelated, three separate models were calculated to prevent multicollinearity.

### MR image acquisition

fMRI data were acquired on a 3 T Siemens Prisma scanner using a T2*-weighted BOLD-sensitive gradient-echo, echo-planar imaging (EPI) sequence [490 volumes/run, 36 oblique axial slices, thickness 2 mm, interslice gap 1 mm, FoV 192 mm, matrix size 64 × 64, in-plane resolution 3 × 3 mm, repetition time (TR) 2000 ms, echo time (TE) 30 ms, flip angle 70°, GRAPPA factor 2]. A T1w structural MR was acquired using a MPRAGE sequence (4:26 min, 192 ascending slices, matrix size 256 × 256, voxel size = 1 × 1 × 1 mm, TR 1900 ms, TE 2.52 ms).

### fMRI pre-processing

The first six volumes of each scan were removed to account for non-steady-state data. Data quality was assessed by visually inspecting reports of the MRI Quality Control Tool (v0.15.2rc1; https://github.com/poldracklab/mriqc; Esteban *et al.*, 2017) for pronounced motion or signal artifacts. Three participants were excluded because their mean framewise displacement (FD; Power *et al.*, 2012) exceeded .25 mm. The remaining participants show sufficient data quality with a mean temporal signal-to-noise ratio of 36.29 (SD 6.04) and a mean FD of .10 mm (SD .05).

Data were preprocessed using fMRIPREP 20.1.1 (Esteban *et al*., 2019). After performing motion estimation, slice time and distortion correction, EPI images were co-registered to the T1w image and normalized to MNI152 space (see the Supplementary Materials for details). Images were then checked for artifacts, brain coverage and correct functional–structural alignment. Subsequent analyses were based on Nipype v1.6.0 (Gorgolewski *et al.*, 2016) using SPM12 v7487 (Welcome Department of Cognitive Neurology, London, http://www.fil.ion.ucl.ac.uk/spm/). As a final step, preprocessed data were smoothed (using a Gaussian kernel with a Full-width at Half-Maximum (FWHM) of 8 mm) to suppress spatial noise.

### fMRI data analysis

As described above, 48 switch and 48 repeat trials were selected for fMRI analyses and modeled as zero-duration stick functions. BOLD responses were modeled with a general linear model (GLM), using a canonical hemodynamic response function with temporal and dispersion derivates and a temporal filter of 128 s. Temporal autocorrelation was estimated using the “FAST” model implemented in SPM (Corbin *et al.*, 2018). Single-subject GLMs included one regressor for each experimental condition (task × switch *vs* repeat). Only correct trials were included. Errors were modeled in an additional regressor of no interest (except for one participant without errors in the selected trials). Six motion parameters (three rotations and translations), FD, the derivative of the RMS variance over voxels (DVARS; Power *et al.*, 2012) and the first six anatomical CompCor regressors (component based noise correction; Behzadi *et al.*, 2007) derived from preprocessing were included to control for motion artifacts and physiological noise, thereby replacing the missing first values of framewise displacement (FD) and DVARS by the mean of the respective variable. Lastly, the following linear contrasts between conditions were estimated for each participant: (i) all switch *vs* all repeat trials (main effect condition), (ii) all emotion *vs* all gender trials (main effect task), (iii) switch trials toward emotion task *vs* repeat trials in emotion task (effect of switching toward the emotion task) and (iv) switch trials toward gender task *vs* repeat trials in gender task (effect of switching away from emotion task).

On the second level, one-sample *t*-tests were performed on each contrast to assess significance of activation differences at the group level. To identify direct associations between switching-related activation and behavioral performance, participants’ RT switch costs (general and asymmetric) were included as between-subjects regressor into the second level analysis of the condition main effect. Furthermore, we tested for neuronal correlates of asymmetric switch costs by calculating a paired *t*-test between the two switch effects, which is technically equivalent to the pre-registered interaction contrast. For evaluation of statistical significance, a cluster-forming voxel-height threshold of *P* < 0.001 was combined with a family-wise error correction at the cluster level to achieve *P* < 0.05 (corrected). Localization of significant clusters was supported by the xjView (https://www.alivelearn.net/xjview) and Anatomy (Eickhoff *et al.*, 2005) toolboxes.

## Results

### Behavioral results

**Fig. 2.**
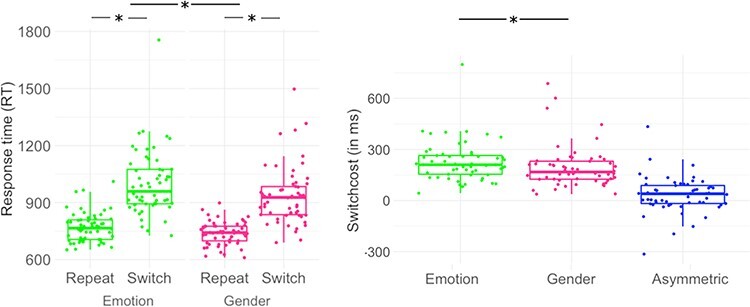
Response times (RT, left) and RT-based switch costs (right) in the affective task-switching paradigm. Significant differences are marked with an asterisk. A metric measure for asymmetric switch costs was calculated by subtracting RT switch cost gender from RT switch cost emotion. One participant showed particularly slow RTs (on average 357 ms slower than the sample mean) and accordingly high switch costs. However, as this individual did not meet the pre-registered accuracy-based criterion for outlier rejection, it remained in the sample. Excluding this participant did not change behavioral results.

ERs were consistently low (see Table 1). In line with previous work (Eckart *et al.*, 2021), internal consistencies of ER were low to moderate with *r*_SB_ = 0.46, 95% CI [0.23, 0.66] in the gender task and *r*_SB_ = 0.64, 95% CI [0.46, 0.77] in the emotion task. In contrast, internal consistencies of RT-based switch costs were consistently high (see Table 1). Repeated-measures ANOVAs on RTs indicated significant main effects of condition, *F*(1,56) = 215.1, *P* < 0.001, and task, *F*(1,56) = 27.97, *P* < 0.001. Responses were faster in repeat trials [EMM = 755 ms, SE ±15.3, 95% CI (724, 785)] than in switch trials [967 ms ± 15.3, 95% CI (937, 998)] and in the gender task [837 ms ± 14.2, 95% CI (809, 866)] than in the emotion task [885 ms ± 14.2, 95% CI (856, 913)]. Moreover, a significant task × condition interaction, *F*(1,56) = 5.49, *P* = 0.02, indicated higher switch costs in the emotion (228.61 ± 117.26 ms; range 42.96–798.43; *t*(81) = -14.22, *P* < 0.001) than in the gender task [195.69 ± 125.39 ms; range 37.38–686.57; *t*(81) = -12.17, *P* < 0.001], *V* = 1177, *P* = 0.005. See Table 1 and Figure 2 for descriptive statistics of mean RTs, effect size estimates and affective switch costs.

**Table 1.** Descriptive statistics for response times (RT) in the affective task-switching paradigm and for the participants’ current mood state

Task measure	Mean	SD	Effect size (Hedges’ *g*)	Reliability (*95% CI)*
*Error rates (ER)*				
Repeat gender	0.07	0.04		
Repeat emotion	0.03	0.03		
Switch to gender	0.06	0.06		
Switch to emotion	0.07	0.07		
*Response times (RT)*				
Repeat gender	739.62	60.46		
Switch to gender	935.30	151.55		
Switch costs gender	195.69	125.39	1.68	0.93 (0.89, 0.96)
Repeat emotion	770.25	77.04		
Switch to emotion	998.86	168.32		
Switch costs emotion	228.61	117.26	1.73	0.92 (0.88, 0.95)
Asymmetric switch costs	32.92	106.05		
*Current mood state*				
PANAS-positive affect	3.12	0.71		0.89 (0.85, 0.93)
PANAS-negative affect	1.25	0.41		0.86 (0.80, 0.91)
STAI sum score	34.52	6.67		0.84 (0.78, 0.89)

Note. PANAS = Positive and Negative Affect Schedule, in repeat trials, we considered the same 48 randomly selected trials that were used as baseline in fMRI contrasts, as those were never directly preceded by a temporal jitter. As reliability measures, we calculated Spearman-Brown corrected correlation (*r*_SB_) for RT-based switch costs and Cronbach’s alpha for questionnaires; 95% confidence intervals are indicated in brackets. The metric measure for asymmetric switch costs was calculated by subtracting the RT switch cost in the gender task from the RT switch cost in the emotion task. See the Methods for details.

Descriptive statistics and reliability measures for participants’ current mood state are presented in Table 1. Cronbach’s *α* of mood state scales were satisfying. When included in the repeated measures ANOVAs, none of the scales had a significant effect on affective task-switching behavior (all *P* ≥ 0.65). Furthermore, none of the mood scales was significantly correlated with RT switch costs (all *P* ≥ 0.1).

### fMRI data

After exclusion of error trials, on average 46.32 ± 1.96 (SD) repeat trials in the emotion task, 44.30 ± 2.12 repeat trials in the gender task, 44.32 ± 3.47 switches to the emotion task and 45.0 ± 2.79 switches to the gender task were included into the fMRI analysis.

#### Main effects of the condition

**Fig. 3.**
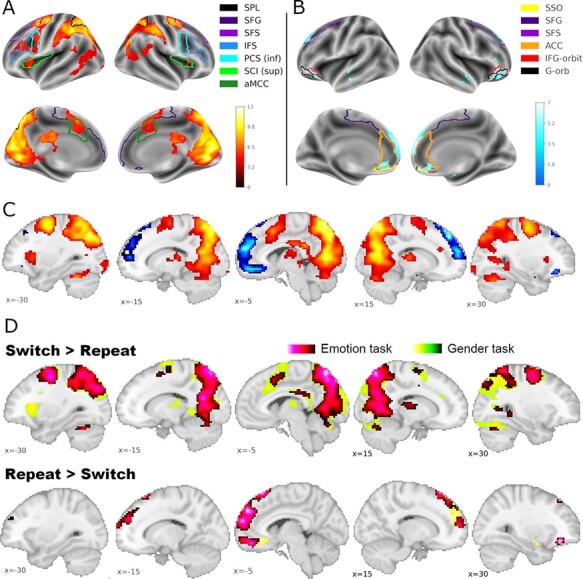
Brain activation patterns for planned contrasts. Brain regions showing significant differences in brain activation and deactivation during switch trials *vs* repeat trials. (A) Increased and (B) decreased brain activation during switch trials, combined across emotion and gender task (main effect condition). (C) Activation patterns for increases and decreases are combined in the slice view. (D) Different distributions of switching-related brain activation patterns (switch vs. repeat) for the two tasks. Activation for the emotion task is shown in the yellow-green color spectrum and for the gender task in the pink-black color spectrum. For more detailed statistical information, see Table 2. Statistical thresholding for (C) and (D) as in (A) and (B). *Note:* For better orientation on the inflated cortex (Panels A and B), selected landmarks from the automatic parcellation with FreeSurfer are given (Destrieux *et al.*, 2010). SPL = superior parietal lobule, SFG = superior frontal gyrus, SFS = superior frontal sulcus, IFS = inferior frontal sulcus, PCS (inf) = inferior part of the precentral sulcus, SCI (sup) = superior segment of the circular sulcus of the insula, aMCC = anterior midcingulate cortex, SSO = Suborbital sulcus, ACC = Anterior part of the cingulate gyrus and sulcus, IFG-orbit = Orbital part of the inferior frontal gyrus; G-orb = orbital gyri. X coordinate refers to MNI coordinates along the sagittal (left/right) axis. Statistical maps are depicted with a voxel-level threshold of *p* = .001 (uncorrected) and a FWE cluster-level correction of *p* < .05.

Enhanced activation on switch as compared to repeat trials was observed in an extended frontal cluster involving the bilateral superior frontal cortex and supplementary motor area (SMA), left middle and inferior frontal gyri including the inferior frontal junction area and the bilateral insulae. Switch-related activity was also seen in an extensive bilateral occipito-parietal cluster and in more focused bilateral clusters in the occipital poles (see Table 2A and hot colors in Figure 3A and C). The reverse contrast, repeat > switch, yielded clusters in bilateral medial frontal and orbitofrontal cortices (extending to the pars triangularis of the inferior frontal gyrus), the left mid-portion of the middle temporal gyrus and right hippocampus (Table 2B; cool colors in Figure 3B and C). These regions showed increased activity during repeat trials in previous investigations of affective task switching (Piguet *et al.*, 2013) and are frequently activated in cognitively low-demanding conditions (Burgess *et al.*, 2007). When switching-related brain activations were calculated separately for each task, very similar activation patterns emerged (Table 2; compare Figures 3D and C).

**Table 2.** Peak activation foci of affective task-switching effects, i.e. activation in switch trials as compared to repeat trials

	Hemisphere	Brodmann area	MNI coordinates (*x*, *y*, *z*)			Cluster extent (voxels)	*Z*
A. Switch ≥ repeat:							
All trials (i.e. combining gender task and emotion task)							
Superior frontal cortex, extending to the precentral cortex (including left middle and inferior frontal cortex with inferior frontal junction, portions of the left insula, bilateral supplemental motor area, medial frontal cortex and the middle part of the cingulum)	R/L	6, 8, 9, 13, 24, 32, 44, 45, 46	**−30** **−**30 26	**−4** **−**10 **−**10	**60** 54 57	**3019**	**7.70** 7.65
							7.48
Insula	R	13	**36**	**20**	**12**	**70**	**4.33**
Occipito-parietal cortex, extending to the post-central gyrus (including the inferior and superior parietal lobule, cuneus, precuneus and thalamus).	R/L	1, 2, 3, 4, 5, 7, 13, 17, 18, 19, 20, 21, 22, 23, 29, 30, 31, 37, 39, 40	**2** **−**4 14	**−72** **−**72 **−**72	**12** 6 60	**11 886**	**>8.00** >8.00
							>8.00
Occipital pole	R	18	**30**	**−100**	**−10**	**62**	**4.82**
	L	18	**−24**	**−106**	**−10**	**126**	**4.72**
Emotion task only							
Superior frontal cortex, extending to the precentral cortex (including middle and inferior frontal cortex with inferior frontal junction, portions of the left insula, orbitofrontal cortex, bilateral supplemental motor area, medial frontal cortex and the middle part of the cingulum)	R/L	6, 8, 9, 13, 24, 32,45, 46, 47	**−28** **−**28 **−**4	**−12** **−**0 14	**54** 60 48	**2537**	**7.11** 6.77
							6.26
Thalamus	L	n/a	**−18** **−**18	**−24** **−**16	**12** 3	**92**	**4.75** 4.57
			**−**10	**−**18	15		4.43
Middle and inferior temporal gyrus	L	20, 37	**−58** **−**42	**−42** **−**64	**−13** **−**25	**124**	**4.00** 3.76
			**−**54	**−**60	**−**16		3.61
Inferior parietal lobule extending to the posterior superior temporal gyrus	R	13, 40	**44** 56	**−46** **−**40	**21** 27	**72**	**4.17** **3.89**
Occipito-parietal cortex, extending to the postcentral gyrus (including the inferior and superior parietal lobule, and precuneus)	R/L	2, 3, 5, 7, 17, 18, 19, 23, 30, 31, 39, 40	**−6** **−**12	**−76** **−**78	**9** 57	**8455**	**>8.00** >8.00
			**−**34	**−**52	36		7.73
Gender task only							
Superior frontal cortex, extending to the precentral cortex (including middle and inferior frontal cortex with inferior frontal junction, bilateral supplemental motor area, medial frontal cortex and the middle part of the cingulum)		6, 9, 24, 32	**−24** 24 26	**−4** **−**4 **−**10	**56** 50 60	**1312**	**7.04** 6.59 6.43
Thalamus (extending to the posterior cingulum)	R/L	n/a	**6** 14	**−22** **−**16	**12** 8	**274**	**5.07** 4.60
			**−**4	**−**28	26		4.49
Inferior parietal lobule extending to the posterior superior temporal gyrus	R	13, 40	**60** 68	**−40** **−**36	**18** 30	**104**	**3.77** 3.68
			48	**−**42	18		3.52
Occipito-parietal cortex, extending to the postcentral gyrus (including the inferior and superior parietal lobule and precuneus)	R/L	1, 2, 3, 4, 5, 7, 17, 18, 19, 21, 22, 23, 30, 31, 37, 39, 40	**−10** **−**18	**−66** **−**64	**60** 18	**8095**	**>8.00** 7.52
			14	**−**70	60		7.40
B. Repeat ≥ switch							
All trials (i.e. combining gender task and emotion task)							
Medial frontal cortex (including frontal pole and portions of the superior frontal cortex)	R/L	6, 8, 9, 10, 11, 25, 32	**6** 8	**56** 42	**12** 54	**2244**	**−6.01** **−**5.97
			8	54	20		**−**5.91
Orbitofrontal cortex extending to the pars triangularis of the inferior frontal gyrus	L	38, 47	**−42** **−**52	**24** 26	**−18** **−**12	**156**	**−5.00** **−**4.22
Middle frontal gyrus (including frontal orbital cortex, pars triangularis of the inferior frontal gyrus and frontal pole)	R	11, 47	**38** 50 38	**36** 32 24	**−12** 12 –24	**294**	**−5.10** **−**4.56 −4.35
Middle temporal gyrus (including superior temporal gyrus and temporal pole)	R	21	**66** 56	**−10** 6	**−12** **−**22	**174**	**−5.15** **−**4.09
	L	21	**−64**	**−16**	**−12**	**164**	**−5.37**
			**−**60	2	**−**22		**−**3.97
			**−**70	**−**28	**−**4		**−**3.80
Hippocampus (including parahippocampal gyrus)	R	n/a	**20**	**−10**	**−16**	**72**	**−5.09**
			26	2	**−**22		**−**3.66
Emotion task only							
Medial frontal cortex (including portions of the superior frontal cortex)	R/L	8, 9, 10	**12** 14	**54** 44	**20** 44	**465**	**−5.29** **−**4.60
			8	38	56		**−**4.23
Inferior frontal gyrus, pars orbitalis	R	47	**56**	**32**	**−4**	**76**	**−4.39**
			56	32	12		**−**3.75
			42	36	–12		**−**3.62
Orbitofrontal cortex	R/L	11	**−6**	**32**	**−12**	**161**	**−4.88**
			6	48	**−**12		**−**3.96
Middle temporal gyrus	R	21	**62**	**−10**	**−12**	**151**	**−5.38**
			60	6	**−**18		**−**3.81
			54	12	**−**24		**−**3.58
Hippocampus (including parahippocampal gyrus)	R	n/a	**20**	**−10**	**−12**	**69**	**−5.19**
			30	**−**0	**−**18		**−**3.49
Gender task only							
Medial frontal cortex (including frontal pole, paracingulate gyrus and portions of the superior frontal cortex)	R/L	6, 8, 9, 10, 11	**−4** **−**4	**54** 60	**36** 20	**1843**	**−5.31** **−**5.02
			**−**6	30	62		**−**4.97
Middle frontal gyrus (including orbitofrontal cortex)	R	11, 47	**32**	**36**	**−16**	**203**	**−5.14**
			48	44	**−**16		**−**4.45
			38	26	**−**22		**−**4.15
Inferior frontal gyrus	R	46	**42**	**26**	**24**	**115**	**−4.43**
Orbitofrontal cortex extending to the temporal pole	L	38, 47	**−42**	**24**	**−22**	**96**	**−4.61**
			**−**52	24	**−**12		**−**4.25
			**−**46	36	**−**10		**−**3.61
Middle temporal gyrus	L	21	**−64**	**−12**	**−12**	**222**	**−4.94**
			**−**54	**−**16	**−**6		**−**4.64
			**−**58	**−**0	**−**24		**−**4.42

Note: Statistical maps were explored with a voxel-level threshold of *P* = 0.001 (uncorrected) and clusters were reported if they survived Family-Wise Error (FWE) cluster-level correction with *P* < 0.05. n/a was indicated if no Brodmann area could be assigned to a cluster. Maxima with highest z score values within a larger cluster are printed in bold.

#### Correlation with switch costs

**Fig. 4.**
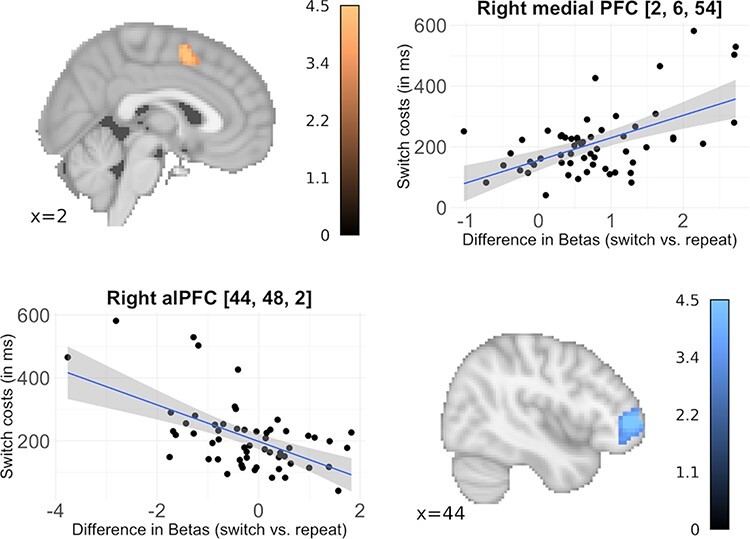
Brain regions showing a direct association between switch-related BOLD activation and behavioral switch costs. A positive association (depicted in the upper left quadrant) was found in medial frontal cortex (located at the border between the rostral cingulate zone and the presupplementary motor cortex), while anterolateral prefrontal cortex showed negative associations (depicted in the lower right quadrant). alPFC = anterolateral prefrontal cortex. Parameter estimates were extracted for the whole cluster and subsequently averaged to get one cluster-specific estimate per participant. Statistical maps are depicted with a voxel-level threshold of *P* = 0.001 (uncorrected) and a FWE cluster-level correction of *P* < 0.05.

Two clusters showed direct associations between the main effect of task switching and RT switch costs (Table 3 and Figure 4): While a positive association was found in the posterior dorsal MFC (roughly at the border between the rostral cingulate zone (RCZ) and pre-SMA (pre-supplementary motor cortex), a cluster in right anterolateral prefrontal cortex (PFC) showed a negative association with behavioral performance (with lower BOLD activation in persons with higher switch costs). Including asymmetric switch costs as a between-subjects covariate in the main effect of task switching yielded no significant clusters.

**Table 3.** Brain regions showing a direct association between switch-related BOLD activation and behavioral switch costs

	Hemisphere	Brodmann area	MNI coordinates (*x*, *y*, *z*)			Cluster extent (voxels)	*Z*
Positive association							
Medial prefrontal cortex (between the rostral cingulate zone and the pre-supplementary motor cortex)	R/L	6, 32	**2** **−**4	**6** 18	**54** 48	**130**	**4.53** 4.11
Negative association							
Anterolateral prefrontal cortex	R	10	**44**	**48**	**2**	**129**	**4.73**

Note: Statistical maps were explored with a voxel-level threshold of *P* = 0.001 (uncorrected) and clusters were reported if they survived FWE cluster-level cluster-level correction with *P* < 0.05. Maxima with highest *z* score values within a larger cluster are printed in bold.

#### Main effect of task

**Fig. 5.**
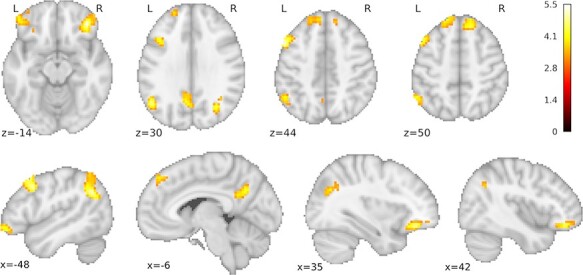
Brain regions showing a main effect of task, i.e. enhanced brain activation in the gender task as compared to the emotion task. Statistical maps are depicted with a voxel-level threshold of *P* = 0.001 (uncorrected) and a FWE cluster-level correction of *P* < 0.05.

Enhanced activation during the gender relative to the emotion task was observed bilaterally in the frontal poles and orbitofrontal as well as superior frontal cortices, in the left middle frontal gyrus, left inferior parietal and right lateral occipital cortex and left precuneus (Table 4, Figure 5). The reverse contrast showed no activations specific to the emotion task.

**Table 4.** Peak activation foci of task effects, i.e. enhanced activations in the gender task as compared to the emotion task

	Hemisphere	Brodmann area	MNI coordinates (*x*, *y*, *z*)			Cluster extent (voxels)	*Z*
Gender task > emotion task							
Orbitofrontal cortex, frontal pole	R	11, 47	**38** 48 38	**38** 50 54	−**12** −10 −10	**109**	**4.91** 3.52 3.28
	L	47	**−48** −40 −34	**48** 38 30	**−10** −16 −18	**86**	**3.96** 3.56 3.46
Superior frontal cortex extending to the frontal pole	R	8	**14** 14 12	**36** 42 24	**50** 42 66	**89**	**4.10** 3.43 3.32
	L	8	**−10** −22	**44** 54	**48** 30	**113**	**4.19** 3.85
			−16	44	24		3.46
Middle frontal gyrus extending to the inferior frontal sulcus	L	8, 9	**−52** −40	**14** 18	**44** 26	**168**	**4.71** 4.06
Precuneus, posterior cingulate cortex	L	7, 31	**−6** −0	**−60** −52	**32** 20	**136**	**3.98** 3.73
Inferior parietal lobe	L	39, 40	**−52**	**−60**	**32**	**180**	**4.66**
			−54	−54	54		4.10
Middle occipital gyrus	R	31	**32**	**−66**	**30**	**63**	**4.15**
			32	−58	32		3.64

Note: Statistical maps were explored with a voxel-level threshold of *P* = 0.001 (uncorrected) and clusters were reported if they survived FWE cluster-level correction with *P* < 0.05. Maxima with highest *z* score values within a larger cluster are printed in bold.

#### Task x condition interaction

**Fig. 6.**
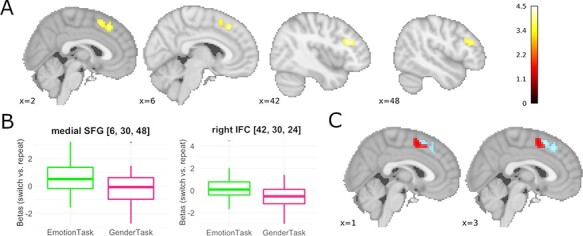
Neuronal representation of asymmetric affective switch costs. (A) Brain regions showing higher activation differences between switch and repeat trials in the emotion task than in the gender task. (B) Comparable to the behavioral switch costs, the activation difference between repeat trials and switch trials (illustrated here on extracted beta values) was higher for the emotion task than for the gender task. (C) Neuroanatomically close but distinct localizations of the medial prefrontal cluster identified as locus of asymmetric switch costs (anterior dorsal MFC involving medial superior frontal gyrus and anterior portions of the pre-SMA, cluster located more anteriorly and depicted in lighter color), and the posterior medial prefrontal cluster whose switch-related activity was directly correlated with behavioral switch costs (RCZ/pre-SMA, cluster located more anteriorly and depicted in darker color). Statistical maps are depicted with a voxel-level threshold of *P* = 0.001 (uncorrected) and a FWE cluster-level correction of *P* < 0.05.

Behavioral results were characterized by an interaction between the task and the condition, reflecting asymmetrically higher switch costs when switching to the emotion task than to the gender task. To determine the neural substrates underlying this pattern, a paired *t*-test was calculated to identify differential switch-related BOLD activation effects between the emotion and the gender task. This analysis identified one cluster in the mid-portion of the right inferior frontal sulcus (extending into middle frontal gyrus) and one cluster in the MFC (more specifically the medial superior frontal gyrus (SFG), posteriorly bordering at the pre-SMA; see Table 5 and Figure 6A). The latter cluster is located directly anterior to the fronto-medial location of the brain-behavior correlation between switch-related activity and behavioral switch costs (Figure 6C). No area showed greater switching activity for the gender task compared to the emotion task.

**Table 5.** Neural regions showing higher activation differences between switch trials and repeat trials in the emotion than in the gender task

	Hemisphere	Brodmann area	MNI coordinates (*x*, *y*, *z*)			Cluster extent (voxels)	*Z*
Inferior frontal cortex	R	9, 46	**42**	**30**	**24**	**64**	**4.06**
Medial superior frontal gyrus (SFG containing parts of the pre-supplementary motor area)	R	6, 8	**6** 2	**30** 14	**48**	**64** 54	**3.88** 3.67

Note: Statistical maps were explored with a voxel-level threshold of *P* = 0.001 (uncorrected), and clusters were reported if they survived FWE cluster-level correction with *P* < 0.05. Maxima with highest *z* score values within a larger cluster are printed in bold.

## Discussion

In this pre-registered fMRI study, we aimed to identify neural activation patterns implicated in affective task switching, i.e. switches between an affective and an affectively neutral (gender) task rule. Replicating previous work (Piguet *et al.*, 2013; Reeck and Egner, 2015), task-switching activity was eminent in a domain-general fronto-parietal network. Activity in posterior MFC (pre-SMA/RCZ) and right anterolateral PFC was directly related to behavioral switch costs. Higher regulatory effort while switching to the emotion task, reflected in asymmetrically higher RT switch costs, was accompanied by increased brain activation in right lateral prefrontal and anterior dorsal MFC, including the medial SFG and anterior parts of the pre-SMA (cp. Johansen-Berg *et al.*, 2004). However, no brain region showed a direct association with a metric measure of asymmetric switch costs. A broadly distributed task-specific activation increase (independent of the switch condition) was evident for the (less dominant) gender task but not for the (more dominant) emotion task.

### Domain-general cognitive control networks regulate affective task switching

The fronto-parietal activation pattern for affective task switching aligns well with established findings for task switching with affectively neutral stimuli (e.g. Kim *et al.*, 2012; Uddin, 2021) and with previous reports on task switching with affective faces (Piguet *et al.*, 2013; Reeck and Egner, 2015). We replicated the latter reports in a substantially larger sample and extended them by examining direct correlations between BOLD activation and behavioral switch costs. Interestingly, the direction of brain-behavior correlations differed between lateral and medial PFC: the anterolateral PFC showed greater recruitment in persons who had lower switch costs. Since anterolateral PFC has specifically been implicated in context-switching (Kim *et al.*, 2012), this result might reflect successful implementation of cognitive control in more efficient task switchers. Posterior MFC activation, on the other hand, showed the opposite pattern, i.e. greater activation associated with less efficient behavioral task switching. This effect is localized in very close proximity to the anterior midcingulate cortex (aMCC) or dorsal anterior part of the cingulate gyrus and sulcus, which is consistently reported as involved in emotional and non-emotional interference processing (Xu *et al.*, 2016) and in studies targeting the negative affect, pain and cognitive control (Shackman *et al.*, 2011) and which was proposed to be involved more generally in the interaction of emotion and cognition (Cromheeke and Mueller, 2014). It was suggested that the RCZ integrates information about pain and potential punishments to signal the amount of adaptive control needed to circumvent negative consequences of a specific situation (Shackman *et al.*, 2011). Accordingly, higher MFC activity during affective task switching could reflect signaling of a higher need for cognitive control in less efficient persons. However, this interpretation needs to be clarified in future research.

### Inhibitory brain circuits are directly related to asymmetric switch costs

Behaviorally, we replicated a previously reported asymmetry with higher RT costs when switching to the emotion as compared to switching to the gender task (Reeck and Egner, 2015; Kraft *et al.*, 2020; Eckart *et al.*, 2021). Higher switch costs in one of two competing task sets have generally been interpreted as indicating differences in task set dominance, with prolonged switch costs when switching to the dominant task (Monsell *et al.*, 2000). Two complementary theoretical models have been proposed to explain this pattern: initially, asymmetric switch costs were mainly attributed to negative priming effects (e.g. Allport *et al.*, 1994), i.e. to an increased effort necessary to release a dominant task set from its greater backward inhibition. Later, the importance of top-down processes and volitional cognitive control when conducting the less dominant task has been emphasized (Yeung and Monsell, 2003). According to this framework, competition between tasks of varying dominance requires increased cognitive control in the less dominant task to assure successful performance, thereby reducing the corresponding switch costs. Supporting this notion, it was reported that behavioral switch costs decreased in affectively neutral tasks when they were paired with an emotion judgment, but increased when mixed with each other (Schuch *et al.*, 2012).

Our study did not identify a brain region whose switching-related activity was (in a correlation analysis) directly associated with a metric measure of asymmetric RT switch cost. However, a paired *t*-test revealed that switching to the more dominant emotion task was associated with increased activity in right inferior PFC and anterior dorsal MFC. These regions are critically involved in inhibitory control (Aron *et al.*, 2014; Cai *et al.*, 2014), including the inhibition of emotional interference (Xu *et al.*, 2016). This supports the assumption that asymmetric behavioral switch costs are—at least partly—a reflection of increased demands on inhibitory control over the previous task set (cp. Reeck and Egner, 2015; Eckart *et al.*, 2021) even though this increased inhibitory control seems not to be directly related to behavioral performance.

Interestingly, this dorsomedial MFC cluster is located dorsally and anterior to the RCZ/pre-SMA cluster showing a condition-independent correlation with behavioral switch costs. Reeck and Egner (2015) reported a main effect of task switching covering both areas (see their Figure 3), and a meta-analysis by Cromheeke and Müller (2014) associated these areas with emotion-cognition interaction in general. However, a recent multivariate pattern analysis of conflict and affect processing provided first evidence suggestive of a dissociation among these MFC subregions: Even though both, conflict and affect, could be decoded from the pre-SMA in that study, only affect could be decoded from more anterior MFC (cf. Figure 4 in Vermeylen *et al.*, 2020). To further explore whether these two adjacent medial frontal regions may indeed dissociate functionally, we conducted a term-based meta-analysis (Yarkoni *et al.*, 2011) for the two MFC clusters identified in the present study (see the Supplementary Materials for details). When leaving aside terms related to neuroanatomy, clearly different cognitive domains, motor acts or specific stimulus types, the remaining set of terms relates both brain regions to motor and executive control processes in general, including concepts like ‘response selection’, ‘execution’, ‘preparatory’, ‘interference’, ‘stop signal’, ‘conflict’, ‘monitoring’ or ‘negative feedback’ (Supplementary Figure S1). However, statistically, the two brain regions did not dissociate, which may be due to the proximity of the two brain regions. This appears consistent with recent work of de la Vega *et al*. (2016) who not only support a functional parcellation of MFC into pre-SMA, posterior and anterior dorsal MFC but also found meta-analytically that all three subregions are involved in cognitive control processes (de la Vega *et al.*, 2016). Thus, in light of our own as well as previous results (Vermeylen *et al.*, 2020), we very tentatively suggest that during affective task switching, the more anterior medial SFG/pre-SMA region might be particularly engaged in inhibitory processes involved in the cognitive control of affective stimuli (cf. Aron *et al.*, 2007; Xu *et al.*, 2016), while the more posteriorly located RCZ/pre-SMA might be a neuronal correlate of the general need for executive control (Shackman *et al.*, 2011; Xu *et al.*, 2016) during task switching. However, this interpretation is speculative and should be replicated and explored more systematically in future research (i.e. by connectivity analyses identifying the exact brain networks with which these regions interact).

### Increased brain activity for the less dominant gender task

Irrespective of the switching condition, increased task-specific activity was observed for the less dominant gender task in a distributed network of orbitofrontal, superior frontal, middle frontal and inferior parietal cortices. While our (considerably more highly powered) study does not replicate the specific region in the right ventrolateral prefrontal cortex (VLPFC) reported by Reeck and Egner (2015), our results are consistent in that task-specific activity is uniquely found for the (less dominant) non-affective task. Affective stimuli disrupt ongoing mental processes and gain privileged access to cognitive resources (e.g. Iordan *et al.*, 2013; Okon-Singer *et al.*, 2015). Accordingly, greater fronto-parietal recruitment during the gender task might indicate the need for suppression of task-irrelevant emotional information (Dolcos *et al.*, 2011; Iordan *et al.*, 2013) when focusing on the task-relevant gender information. This finding is also in line with the above-discussed ‘top-down’ model of asymmetric switch costs (Yeung and Monsell, 2003), which assumes higher amounts of executive control when performing the less dominant task. At the same time, asymmetric switch costs were accompanied by increased activity in inhibitory circuits (see also above). Accordingly, at least in affective task switching, asymmetric switching behavior cannot clearly be attributed to either increased cognitive control exerted on the less-dominant task or increased effort necessary to overcome backward inhibition while switching to the dominant task—but might rather be caused by an interplay of both (as also suggested by Yeung and Monsell, 2003). We propose that future research should elucidate these processes in more detail, e.g. by including affectively neutral control conditions (cp. Schuch *et al.*, 2012).

### Theoretical implications

In the present study, we replicated and extended previous results on affective task switching in a substantially (i.e. almost three times) larger sample. This allowed for statistically well-powered replications of important effects (like asymmetric switch costs) and yielded a more comprehensive understanding of fundamental aspects of the present paradigm, like task-associated activation differences (cp. our Figure 5  *vs*  Table 2 in Reeck and Egner, 2015). This larger sample further allowed us to directly target brain-behavior associations, as well as the neural mechanisms underlying asymmetric affective switch costs. Our data suggest that affective task switching is controlled by a rather domain-general network that is equally implicated in non-affective task switching (e.g. Kim *et al.*, 2012; Richter and Yeung, 2014), while no pronounced involvement of limbic brain areas emerged. Still, studying task switching with affective stimuli and tasks adds important additional dimensions: for example, face stimuli can be classified according to multiple dimensions (i.e. gender, emotion, age, familiarity, etc.) of potentially different behavioral salience and thus task-set dominance (Leleu *et al.*, 2012). Importantly, ‘affective asymmetric switch costs’ in this context cannot be attributed to mere differences in task difficulty: Asymmetrically increased switch costs typically emerge when switching to the easier task (e.g., Yeung and Monsell, 2003; Schneider and Anderson, 2010). However, we here observe the reverse pattern with higher switch costs in the emotion task, whereas the gender task was characterized by generally faster response behavior (see discussion above). This makes differences in task salience or dominance between affective (emotion) and non-affective (gender) facial features the more likely explanation. Future studies should investigate how executive control systems deal with competing information of differing behavioral relevance.

## Conclusion

While increased activity in anterolateral PFC was indicative of efficient task switching, increased activity in posterior dorsal MFC (RCZ/pre-SMA) showed the opposite pattern, probably signaling a higher need for cognitive control. A more dorsally and anteriorly located medial SFG/pre-SMA region was—together with right inferior frontal cortex—particularly engaged during asymmetrically increased switch costs, thus being a potential neuronal correlate of the increased effort while releasing a dominant, emotional task rule from its backward inhibition. Independent of the switching condition, we observed increased activations in lateral prefrontal and parietal cortices during the less-dominant gender task. We propose that this reflects increased volitional top-down control while shielding the less dominant task from task-irrelevant emotional information present in all stimuli. Together, these results suggest a cognitive control network consisting of pre-SMA, medial SFG and middle and anterior aspects of right lateral PFC as being particularly relevant for successful executive control over affective materials. Given that many psychiatric conditions are characterized by disturbed cognitive control over affective information, our paradigm offers a potentially valuable approach to investigate the causes of these disturbances in clinical conditions.

## Supplementary Material

nsac054_Supp

## Data Availability

The data underlying this article will be shared on reasonable request to the corresponding author.
