# Supplementary material for: Neural correlates of affective task switching and asymmetric affective task switching costs
Source: Soc Cogn Affect Neurosci. 2022 Oct 13;18(1):nsac054. doi: 10.1093/scan/nsac054 (PMC9949498; doi:10.1093/scan/nsac054)
Supplement: nsac054_Supp [file nsac054_supp.zip › scan-21-225-File008.docx]

**Supplementary material**

for

**Neural correlates of affective task-switching and asymmetric affective task switching costs**

Cindy Eckart^1^, Dominik Kraft^1^, Lena Rademacher^1, 2^, Christian J. Fiebach^1, 3^

^1^ Department of Psychology, Goethe University Frankfurt, Frankfurt am Main, Germany

^2^ Department of Psychiatry and Psychotherapy, University of Lübeck, Lübeck, Germany

^3^ Brain Imaging Center, Goethe University Frankfurt, Frankfurt am Main, Germany

**Address for Correspondence:**

Dr. Cindy Eckart

Department of Psychology, Goethe University Frankfurt, Theodor-W.-Adorno-Platz 6, Frankfurt am Main 60323, Germany

Electronic address: eckart@psych.uni-frankfurt.de

**Supplemental File:**

- **Supplemental Results:** Comparison of behavioral analyses conducted on the full set of trials instead of the trial selection reported in the main text
- **Supplemental Methods: (f)MRI preprocessing**
- **Supplemental Methods & Results: Term-based meta-analysis of posterior vs. anterior dorsal medial PFC**
- **Supplemental References**

**Supplemental Results: Behavioral re-analyses of the data considering the full set of repeat trials.**

When considering the full set of repeat trials (as opposed to the 48 trials selected for fMRI analysis; cf. Methods of main text), error rates did not change substantially, i.e., .06 ± .03 in gender repeat trials (as opposed to .07 ± .04 in the selected trials) and .05 ± .04 for emotion repeat trials (as opposed to .03 ± .03 in selected trials). In a direct comparison, no significant difference between ERs in selected vs. non-selected repeat trials emerged, *V* = 946, *p* = .08 (Wilcoxon signed-rank test).

Response times (RT) were substantially slowed down when considering the full set of repeat trials, i.e., 799.59 ± 76.55ms for gender repeat trials (as opposed to 739.62 ± 60.46ms in the selected trials as described in the manuscript) and 831.76 ± 89.48 for emotion repeat trials (as opposed to 770.25 ± 77.04ms in selected trials). In a direct comparison, RTs indeed differed significantly between selected and non-selected repeat trials, *V* = 1653, *p* < .001. See Wodka *et al.* (2009) for a discussion of possible causes of these differences).

However, main statistical results were not affected by this deceleration and the repeated measures ANOVA continues to indicate significant main effects of condition, *F*(1,56) = 163.2, *p* < .001, and task, *F*(1,56) = 23.83, *p* < .001. Comparable to the analysis of selected trials (cf. results reported in the main text), responses were generally faster in repeat trials (estimated marginal mean (*EMM)* = 816ms, *SE* ±16, 95% CI [784, 847]) as compared to switch trials (967ms ± 16, 95% CI [935, 999]) and in the gender task (867ms ± 15.6, 95% CI [836, 899]) as compared to the emotion task (915ms ± 15.6, 95% CI [884, 946]). Moreover, a significant task x condition interaction, *F*(1,56) = 8.68, *p* = .005, indicated higher switch costs in the emotion (mean = 167.10 ± 99.11ms; range 17.38 – 639.66; cp: 228.61 ± 117.26ms in selected trials) than in the gender task (135.71 ± 97.09ms; range -20.42 – 527.09; cp.: 195.69 ± 125.39ms in selected trials), *V* = 1220, *p* = .002. See main text for ANOVA results based on the selected repeat trials.

**Supplemental Methods: MRI Data Preprocessing**

FMRI data were organized according to the Brain Imaging Data Structure (BIDS; Gorgolewski et al., 2016) after reconstruction and preprocessing was performed using fMRIPrep 20.1.1 (Esteban et al., 2018a, 2018b; RRID:SCR_016216) based on Nipype 1.5.0 (Gorgolewski et al., 2011; Gorgolewski et al., 2018; RRID:SCR_002502). Within its functional processing workflow, many internal operations of fMRIPrep use Nilearn 0.6.2 (Abraham et al. 2014, RRID:SCR_001362). For more details of the pipeline, see [the section corresponding to workflows in fMRIPrep’s documentation](https://fmriprep.readthedocs.io/en/latest/workflows.html) (https://fmriprep.org/en/latest/workflows.html). The following paragraphs are based on a ‘boilerplate’ text (released under CC0 license) automatically generated by *fMRIprep* and describe the preprocessing in more detail. The text was manually edited for syntax and readability.

***Preprocessing of Anatomical MRI Data***

One T1-weighted (T1w) image was found within the input BIDS dataset. The T1-weighted (T1w) image was corrected throughout the workflow. The T1w-reference was then skull-stripped with a Nipype implementation of the antsBrainExtraction.sh workflow (from ANTs), using OASIS30ANTs as target template. Brain tissue segmentation of cerebrospinal fluid (CSF), white-matter (WM), and gray-matter (GM) was performed on the brain-extracted T1w using *FAST* (FSL 5.0.9, RRID:SCR_002823, Zhang, Brady, and Smith 2001). Brain surfaces were reconstructed using *recon-all* (FreeSurfer 6.0.1, RRID:SCR_001847, Dale, Fischl, and Sereno 1999), and the brain mask estimated previously was refined with a custom variation of the method to reconcile ANTs-derived and FreeSurfer-derived segmentations of the cortical gray-matter of Mindboggle (RRID:SCR_002438, Klein et al. 2017). Volume-based spatial normalization to the *ICBM 152 Nonlinear Asymmetrical template version 2009c* (Fonov et al., 2009, RRID:SCR_008796; TemplateFlow ID: MNI152NLin2009cAsym) standard space was performed through nonlinear registration with *antsRegistration* (ANTs 2.2.0), using brain-extracted versions of both T1w reference and the T1w template. for intensity non-uniformity (INU) with *N4BiasFieldCorrection* (Tustison et al. 2010), distributed with *ANTs 2.2.0* (Avants et al. 2008, RRID:SCR_004757), and used as T1w-reference

***Preprocessing of Functional MRI Data***

For each of the 2 BOLD runs found per subject, the following preprocessing was performed. First, a reference volume and its skull-stripped version were generated using a custom methodology of fMRIPrep. Head-motion parameters with respect to the BOLD reference (transformation matrices, and six corresponding rotation and translation parameters) are estimated before any spatiotemporal filtering using *MCFLIRT* (FSL 5.0.9, Jenkinson et al. 2002). BOLD runs were slice-time corrected using 3dTshift from *AFNI* *20160207* (Cox and Hyde 1997, RRID:SCR_005927). A B0-nonuniformity map was estimated based on echo-planar imaging (EPI) references with opposing phase-encoding directions, with *3dQwarp* Cox and Hyde (1997; AFNI 20160207). Based on the estimated susceptibility distortion, a corrected EPI (echo-planar imaging) reference was calculated for a more accurate co-registration with the anatomical reference.

The BOLD reference was then co-registered to the T1w reference using FreeSurfer’s *bbregister* which implements boundary-based registration (Greve and Fischl 2009). Co-registration was configured with six degrees of freedom. The BOLD time-series (including slice-timing correction) were resampled onto their original, native space by applying a single, composite transform to correct for head-motion and susceptibility distortions. These resampled BOLD time-series will in the following be referred to as preprocessed BOLD in original space, or just preprocessed BOLD. The BOLD time-series were resampled into standard space, generating a preprocessed BOLD run in MNI152NLin2009cAsym space. First, a reference volume and its skull-stripped version were generated using a custom methodology of fMRIPrep.

Several confounding time-series were calculated based on the preprocessed BOLD: framewise displacement (FD), derivative of RMS variance over voxels (DVARS), and three region-wise global signals. FD was computed using two formulations following Power (absolute sum of relative motions, Power et al. (2014) and Jenkinson (relative root mean square displacement between affines, Jenkinson et al. (2002). FD and DVARS are calculated for each functional run, both using their implementations in Nipype (following the definitions by Power et al., 2014). The three global signals are extracted within the CSF, the WM, and the whole-brain masks. Additionally, a set of physiological regressors were extracted to allow for component-based noise correction (CompCor, Behzadi et al. 2007): Principal components are estimated after high-pass filtering the preprocessed BOLD time-series (using a discrete cosine filter with 128s cut-off) for the two CompCor variants, i.e., temporal (tCompCor) and anatomical (aCompCor). tCompCor components are then calculated from the top 5% variable voxels within a mask covering the subcortical regions. This subcortical mask is obtained by heavily eroding the brain mask, which ensures it does not include cortical GM regions. For aCompCor, components are calculated within the intersection of the aforementioned mask and the union of CSF and WM masks calculated in T1w space, after their projection to the native space of each functional run (using the inverse BOLD-to-T1w transformation). Components are also calculated separately within the WM and CSF masks. For each CompCor decomposition, the k components with the largest singular values are retained, such that the retained components’ time series are sufficient to explain 50 percent of variance across the nuisance mask (CSF, WM, combined, or temporal). The remaining components are dropped from consideration. The head-motion estimates calculated in the correction step were also placed within the corresponding confounds file. The confound time series derived from head motion estimates and global signals were expanded with the inclusion of temporal derivatives and quadratic terms for each (Satterthwaite et al. 2013). Frames that exceeded a threshold of 0.5 mm FD or 1.5 standardised DVARS were annotated as motion outliers (*Note*: In this analysis a specific FD threshold of .25 was chosen for data exclusion). All resamplings can be performed with a single interpolation step by composing all the pertinent transformations (i.e., head-motion transform matrices, susceptibility distortion correction, and co-registrations to anatomical and output spaces). Gridded (volumetric) resamplings were performed using *antsApplyTransforms* (ANTs), configured with Lanczos interpolation to minimize the smoothing effects of other kernels (Lanczos 1964). Non-gridded (surface) resamplings were performed using mri_vol2surf (FreeSurfer).

**Supplemental Methods and Results: Term-based meta-analysis of posterior vs. anterior dorsal medial PFC**

A term-based meta-analysis was conducted based on the *neurosynth* database (v 0.7; Yarkoni et al., 2011; see also https://neurosynth.org/). Regions of interest (ROIs) were the two medial PFC clusters visualized in Figure 6C, i.e., posterior dorsal MFC (involving the rostral cingulate zone/RCZ and pre-SMA) and anterior dorsal MFC (i.e., the medial portion of the superior frontal gyrus and the anterior-most part of the pre-SMA). Functional ROIs were determined by thresholding statistical parametric maps as described in the main text. The version of the neurosynth database used for the present analysis includes 14,371 fMRI studies, peak coordinates for 507,891 activation peaks (in MNI152 space), as well as frequencies of occurrences of all words used in the abstracts of those studies. For each ROI, studies were grouped into those that reported a respective activation and those that did not based on a threshold of at least 5% activated voxels. Subsequently, a naive Bayes classifier was trained to distinguish these groups of studies and functional preference profiles were defined through term-specific log odds ratios (LOR) obtained from fitted models, via extracting the log of the ratio of a term being present in activating studies and being present in not-activating studies. To assess possible functional specializations, all terms related to neuroanatomy, clearly different cognitive domains (e.g., calculation, motor imagery), motor acts (e.g., finger tapping, saccade), or specific stimulus types (e.g., word pairs) were filtered out and remaining terms were ranked according to the LOR. The statistical significance of each term’s predictive ability for each ROI was computed through permutation tests. Based on 1,000 group-label (i.e., activating, not-activating study) permutations and respective LOR extractions, a null distribution was obtained against which the initial LOR could be tested. The resulting p-values were corrected for multiple comparisons through FDR with a threshold of q < 0.01. Lastly, LORs were compared between the two ROIs, to determine whether different cognitive terms were differentially associated with anterior vs. posterior MFC. To this end, bootstrapping with replacement was applied to extract LORs for each ROI 1,000 times to then compare the respective 95% CI of a term between ROIs. Supplementary Figure S1 shows terms with highest LORs per region, after filtering out duplicates and all anatomical terms.

| 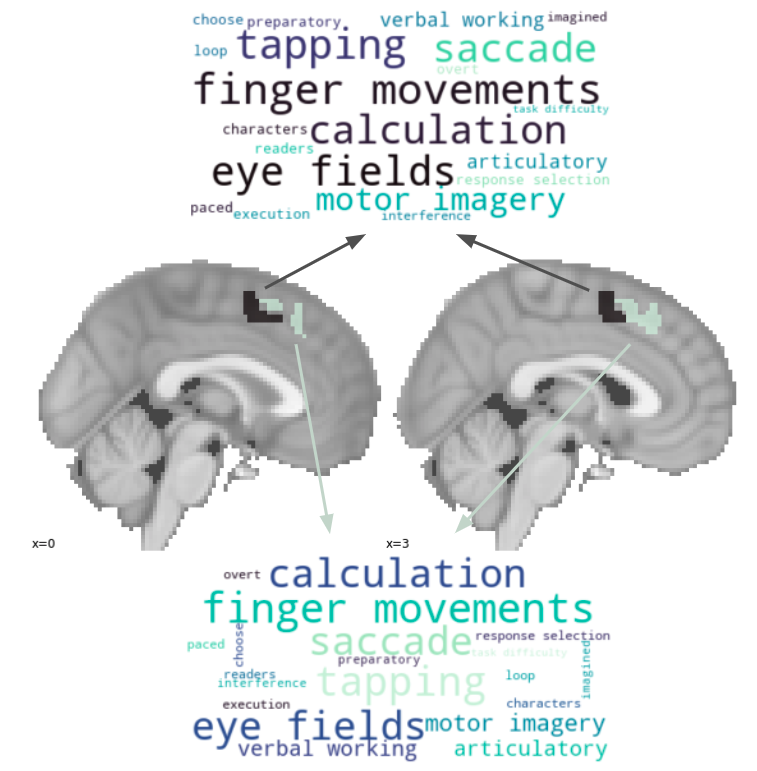 |  |
| --- | --- |
| **Supplementary Figure S1.** Term-based meta-analysis of posterior vs. anterior medial PFC. Word cloud of terms associated with posterior medial PFC (RCZ/pre-SMA; black cluster) vs. anterior medial PFC (medial superior frontal gyrus and pre-SMA; grey cluster). Region of interest clusters were resampled from the results maps reported in the main text (cp. Figure 6C). |  |

### Supplemental References

Abraham, Alexandre, Fabian Pedregosa, Michael Eickenberg, Philippe Gervais, Andreas Mueller, Jean Kossaifi, Alexandre Gramfort, Bertrand Thirion, and Gael Varoquaux. 2014. “Machine Learning for Neuroimaging with Scikit-Learn.” Frontiers in Neuroinformatics 8. <https://doi.org/10.3389/fninf.2014.00014>.

Avants, B.B., C.L. Epstein, M. Grossman, and J.C. Gee. 2008. “Symmetric Diffeomorphic Image Registration with Cross-Correlation: Evaluating Automated Labeling of Elderly and Neurodegenerative Brain.” Medical Image Analysis 12 (1): 26–41. <https://doi.org/10.1016/j.media.2007.06.004>.

Behzadi, Yashar, Khaled Restom, Joy Liau, and Thomas T. Liu. 2007. “A Component Based Noise Correction Method (CompCor) for BOLD and Perfusion Based fMRI.” NeuroImage 37 (1): 90–101. <https://doi.org/10.1016/j.neuroimage.2007.04.042>.

Cox, Robert W., and James S. Hyde. 1997. “Software Tools for Analysis and Visualization of fMRI Data.” NMR in Biomedicine 10 (4-5): 171–78. [https://doi.org/10.1002/(SICI)1099-1492(199706/08)10:4/5<171::AID-NBM453>3.0.CO;2-L](https://doi.org/10.1002/(SICI)1099-1492(199706/08)10:4/5%3c171::AID-NBM453%3e3.0.CO;2-L).

Dale, Anders M., Bruce Fischl, and Martin I. Sereno. 1999. “Cortical Surface-Based Analysis: I. Segmentation and Surface Reconstruction.” NeuroImage 9 (2): 179–94. <https://doi.org/10.1006/nimg.1998.0395>.

Vega, A. de la, Chang, L.J., Banich, M.T., et al. (2016). Large-Scale Meta-Analysis of Human Medial Frontal Cortex Reveals Tripartite Functional Organization. *Journal of Neuroscience*, **36**, 6553–62. https://doi.org/10.1523/JNEUROSCI.4402-15.2016.

Esteban, Oscar, Ross Blair, Christopher J. Markiewicz, Shoshana L. Berleant, Craig Moodie, Feilong Ma, Ayse Ilkay Isik, et al. 2018. “FMRIPrep.” Software. Zenodo. <https://doi.org/10.5281/zenodo.852659>.

Esteban, Oscar, Christopher Markiewicz, Ross W Blair, Craig Moodie, Ayse Ilkay Isik, Asier Erramuzpe Aliaga, James Kent, et al. 2018. “fMRIPrep: A Robust Preprocessing Pipeline for Functional MRI.” Nature Methods. <https://doi.org/10.1038/s41592-018-0235-4>.

Fonov, VS, AC Evans, RC McKinstry, CR Almli, and DL Collins. 2009. “Unbiased Nonlinear Average Age-Appropriate Brain Templates from Birth to Adulthood.” NeuroImage 47, Supplement 1: S102. <https://doi.org/10.1016/S1053-8119(09)70884-5>.

Glasser, Matthew F., Stamatios N. Sotiropoulos, J. Anthony Wilson, Timothy S. Coalson, Bruce Fischl, Jesper L. Andersson, Junqian Xu, et al. 2013. “The Minimal Preprocessing Pipelines for the Human Connectome Project.” NeuroImage, Mapping the connectome, 80: 105–24. <https://doi.org/10.1016/j.neuroimage.2013.04.127>.

Gorgolewski, K., C. D. Burns, C. Madison, D. Clark, Y. O. Halchenko, M. L. Waskom, and S. Ghosh. 2011. “Nipype: A Flexible, Lightweight and Extensible Neuroimaging Data Processing Framework in Python.” Frontiers in Neuroinformatics 5: 13. <https://doi.org/10.3389/fninf.2011.00013>.

Gorgolewski, Krzysztof J., Oscar Esteban, Christopher J. Markiewicz, Erik Ziegler, David Gage Ellis, Michael Philipp Notter, Dorota Jarecka, et al. 2018. “Nipype.” Software. Zenodo. <https://doi.org/10.5281/zenodo.596855>.

Greve, Douglas N, and Bruce Fischl. 2009. “Accurate and Robust Brain Image Alignment Using Boundary-Based Registration.” NeuroImage 48 (1): 63–72. <https://doi.org/10.1016/j.neuroimage.2009.06.060>.

Jenkinson, Mark, Peter Bannister, Michael Brady, and Stephen Smith. 2002. “Improved Optimization for the Robust and Accurate Linear Registration and Motion Correction of Brain Images.” NeuroImage 17 (2): 825–41. <https://doi.org/10.1006/nimg.2002.1132>.

Klein, Arno, Satrajit S. Ghosh, Forrest S. Bao, Joachim Giard, Yrjö Häme, Eliezer Stavsky, Noah Lee, et al. 2017. “Mindboggling Morphometry of Human Brains.” PLOS Computational Biology 13 (2): e1005350. <https://doi.org/10.1371/journal.pcbi.1005350>.

Lanczos, C. 1964. “Evaluation of Noisy Data.” Journal of the Society for Industrial and Applied Mathematics Series B Numerical Analysis 1 (1): 76–85. <https://doi.org/10.1137/0701007>.

Power, Jonathan D., Anish Mitra, Timothy O. Laumann, Abraham Z. Snyder, Bradley L. Schlaggar, and Steven E. Petersen. 2014. “Methods to Detect, Characterize, and Remove Motion Artifact in Resting State fMRI.” NeuroImage 84 (Supplement C): 320–41. <https://doi.org/10.1016/j.neuroimage.2013.08.048>.

Satterthwaite, Theodore D., Mark A. Elliott, Raphael T. Gerraty, Kosha Ruparel, James Loughead, Monica E. Calkins, Simon B. Eickhoff, et al. 2013. “An improved framework for confound regression and filtering for control of motion artifact in the preprocessing of resting-state functional connectivity data.” NeuroImage 64 (1): 240–56. <https://doi.org/10.1016/j.neuroimage.2012.08.052>.

Tustison, N. J., B. B. Avants, P. A. Cook, Y. Zheng, A. Egan, P. A. Yushkevich, and J. C. Gee. 2010. “N4ITK: Improved N3 Bias Correction.” IEEE Transactions on Medical Imaging 29 (6): 1310–20. <https://doi.org/10.1109/TMI.2010.2046908>.

Wodka, E.L., Simmonds, D.J., Mahone, E.M., et al. (2009). Moderate variability in stimulus presentation improves motor response control. *Journal of clinical and experimental neuropsychology*, 31, 483–88. https://doi.org/10.1080/13803390802272036.

Yarkoni, T., Poldrack, R.A., Nichols, T.E., et al. (2011). Large-scale automated synthesis of human functional neuroimaging data. *Nature Methods*, 8, 665–70. https://doi.org/ 10.1038/nmeth.1635.

Zhang, Y., M. Brady, and S. Smith. 2001. “Segmentation of Brain MR Images Through a Hidden Markov Random Field Model and the Expectation-Maximization Algorithm.” IEEE Transactions on Medical Imaging 20 (1): 45–57. <https://doi.org/10.1109/42.906424>.
